# Metabolomics Combined with Photosynthetic Analysis Reveals Potential Mechanisms of Phenolic Compound Accumulation in *Lonicera japonica* Induced by Nitrate Nitrogen Supply

**DOI:** 10.3390/ijms26094464

**Published:** 2025-05-07

**Authors:** Yiwen Cao, Yating Yang, Zhengwei Tan, Xihan Feng, Zhiyao Tian, Tianheng Liu, Yonghui Pan, Min Wang, Xiaoyu Su, Huizhen Liang, Shiwei Guo

**Affiliations:** 1 Jiangsu Provincial Key Laboratory for Solid Organic Waste Utilization, Key Laboratory of Organic-Based Fertilizers of China, Jiangsu Collaborative Innovation Center for Solid Organic Wastes, Nanjing Agricultural University, Weigang 1, Nanjing 210095, China; 2020203071@stu.njau.edu.cn (Y.C.);; 2 Institute of Chinese Herbal Medicines, Henan Academy of Agricultural Sciences, Zhengzhou 450002, Chinasuxiaoyu_2014@163.com (X.S.); 3 Provincial Key Laboratory of Conservation and Utilization of Traditional Chinese Medicine Resources, Zhengzhou 450002, China

**Keywords:** nitrate, caffeoylquinic acids, phenolic compounds, mesophyll conductance, photosynthesis, *Lonicera japonica*

## Abstract

Mineral nutrition is of vital importance in plant growth and secondary metabolites accumulation, and thereby in the nutritional value of plants. In *Lonicera japonica*, a preference to nitrate (NO_3_^−^−N) in comparison to ammonium (NH_4_^+^−N) was found in our previous study, which can be revealed from the rapid growth rate of *L. japonica* under NO_3_^−^−N. This study assessed whether a preference for nitrogen sources could invoke metabolic reprogramming and interrelationships between factors. NO_3_^−^−fed plants exhibited substantial enhancement of carbon stimulation, which was strongly and positively correlated with mesophyll conductance. As a result, the elevated carbon flux by NO_3_^−^ supplement was shuttled to phenolic metabolites synthesis, including flavones and caffeoylquinic acids compounds. Notably, the stimulation was triggered by changes in the NO_3_^−^ and C/N ratio and was mediated by the induction of several enzymes in the phenylpropanoid pathway. On the contrary, NH_4_^+^ plants showed an increment in the content of nitrogen, carbohydrates, and amino acids (mainly a strong increase in citrulline and theanine). Within secondary metabolism, NH_4_^+^ may involve active lignin metabolism, showing a dramatic increment in hydroxy−ferulic acid and lignin content. This work provides significant insights regarding the mechanisms of *L. japonica* in response to diverse nitrogen regimes and effective strategies of nitrogen fertilizer input for *L. japonica*.

## 1. Introduction

Caprifoliaceae, a plant family of dicotyledons, comprises ~800 species with worldwide distribution [1]. *Lonicera japonica*, commonly known as honeysuckle, is perhaps the most popular plant in the Caprifoliaceae family. The pharmacopeia of countries, such as China and Japan, has recorded the medicinal applications of this species [2], which include treating different types of viral infections, such as SARS coronavirus, H1N1 flu virus, and the influenza A virus [3]. Additionally, the active compounds extracted from *L. japonica* have extensive worldwide applications in industries such as cosmetics, pharmaceuticals, and food [4]. Improving the growth of *L. japonica*, especially the accumulation of bioactive substances, would be a key approach in securing commercial *L. japonica* productivity.

Phytochemical studies have shown that *L. japonica* has a complex composition of secondary substances, including flavonoids and (poly)phenolic acids, which are responsible for its pharmacological activity and nutritional values [2,4]. In particular, the abundant chlorogenic acid (CGA) and its derivatives are important phenolic components in *L. japonica*, playing a vital role in pharmacological activities [5]. Phenolic metabolites are the most prevalent secondary substances in plants, and researchers have recently focused significant attention on studying them [6,7,8]. Previous studies have extensively examined the effects of the variability of environmental factors, e.g., salt [9], drought [10], nutrition [11], CO_2_ concentration [12], and light sources [13], on the accumulation of phenolic compounds.

During these episodes, nitrogen (N) serves as an indispensable nutrient and is available to plants mainly in the forms of nitrate (NO_3_^−^) and ammonium (NH_4_^+^) in agricultural soils [14]. NO_3_^−^ is the primary form of N utilized by plants. Although ammonium can also be absorbed, sole NH_4_^+^ application is usually detrimental to plant growth and disrupts metabolism [15]. The N assimilation characteristics of plants exhibit notable differences when N is provided in the form of NO_3_^−^ or NH_4_^+^. NO_3_^−^ is first reduced to NH_4_^+^ and then incorporated into amino acids through catalysis by glutamine synthetase/glutamate synthase (GS/GOGAT). In this context, utilization of NO_3_^−^ or NH_4_^+^ is represented by quite different demands for energy and carbon (C) skeletons [16]. Moreover, transformations between N− and C−based metabolites are commonly observed. The C−based substances primarily consist of phenolics, e.g., phenolic acids, flavonoids, and anthocyanins [17].

Agricultural practices through the choice of N source have been found to have considerable diverse effects on the phenolic substance. Generally, woody plants showed a better growth performance in a condition of nitrite, i.e., a study on *Cyclocarya paliurus* showed that sole NO_3_^−^ supplementation significantly improved the accumulation of polyphenolic compounds [18]. With regard to herbaceous plants, Lang et al. [19] observed that phenolic acids in grape leaves were significantly altered by fertilization with different N forms, and nitrate application largely improved phenolic acid content. Similarly, Zhu et al. [20] found that a NH_4_^+^/NO_3_^−^ ratio of 1:3 resulted in the highest accumulation of rosmarinic acid and flavonoids. On the contrary, Prinsi et al. [21] showed an increased content of phenolic acid in the condition of NH_4_^+^. Moreover, the phenolic compound biosynthesis pathways were regulated by the N form at the gene level. Based on transcriptome profiles of *Camellia sinensis*, it was shown that chalcone isomerase and chalcone synthase were induced in plants supplied with NO_3_^−^ in comparison with NH_4_^+^ [22].

According to Domínguez et al. [23], the impact of N source on the phenolics accumulation depended largely on plant preference for N forms. For instance, NH_4_^+^-sensitive spinach exhibited increased levels of total soluble phenol in shoots, while no significant elevation was observed in ammonium-tolerant peas when comparing plants grown with NO_3_^−^ and those grown with NH_4_^+^. N form can also regulate phenolic metabolic processes by influencing the synthesis of precursor amino acids and carbon–nitrogen metabolism, or as a signal transmitter. Aromatic amino acids are especially closely related to the synthesis of phenolic metabolites [24]. Li et al. [25] found that the increase in CO_2_ led to a decrease in the contents of phenylalanine and tyrosine in strawberries, thereby reducing the accumulation of metabolites such as anthocyanins, eugenol, and lignin. Moreover, the carbon–nutrition balance (CNB) hypothesis is often applied to explain the influence of N availability on plant phenolic compounds accumulation [26]. Based on the CNB hypothesis, the biosynthesis of phenolics is constrained by N availability and net photosynthetic rate (*P*_n_) in plants, e.g., the reduced N content of leaf tissues; additionally, a considerable *P*_n_ content would increase phenolics content. In agreement with this, a recent study on *Andrographis paniculata* suggested that the accumulation of carbohydrates, *P*_n_, and photosynthetic N use efficiency largely stimulates the biosynthesis of andrographolide (C−based secondary metabolites) [27]. In this context, a comprehensive investigation of metabolic profiles when exposed to different N forms could provide valuable insights.

A previous study of *L. japonica* showed a preference for NO_3_^−^ over NH_4_^+^, indicated by the rapid growth rate and higher photosynthetic efficiency under NO_3_^−^ nutrition [28]. However, it is not sufficiently known how the increased photosynthesis under NO_3_^−^ conditions affects leaf metabolism or, especially, the biosynthesis of phenolic metabolites (CGA, 3,5−di−caffeoylquinic acid (3,5−di−CQA) and 4,5−di−caffeoylquinic acid (4,5−di−CQA)) in *L. japonica*. Therefore, a hypothesis was proposed that the increased C/N ratio under NO_3_^−^ conditions might induce the accumulation of specialized phenolic components in *L. japonica* compared with NH_4_^+^. The availability of omics in conjunction with the basic phenotype, such as photosynthetic parameters, helps to facilitate a more nuanced analysis [29,30,31]. The research findings can provide a scientific basis for enhancing *L. japonica* growth so that it can be produced in agricultural settings.

## 2. Results

### 2.1. Plant Growth and Physiology

In the present study, *L. japonica* grown in NH_4_^+^ nutrition showed significant growth depression. Sole NO_3_^−^ and mixed N treatments significantly increased leaf biomass and C accumulation in comparison with sole NH_4_^+^ (Table 1), but had little effect on the area of the newly expanded leaf. On the contrary, the leaf N content was greater in the presence of NH_4_^+^, with an approximately 14% increase compared with sole NO_3_^−^ treatment. Further analysis revealed no significant difference in NH_4_^+^ content among the treatments, while NO_3_^−^ content was higher in the sole NO_3_^−^ treatment compared with the mixed N and sole NH_4_^+^ treatments (Table 1), suggesting a higher N turnover in the presence of NH_4_^+^ nutrition. As a result, the C/N ratio was statistically different among the treatments.

**Figure 1:**
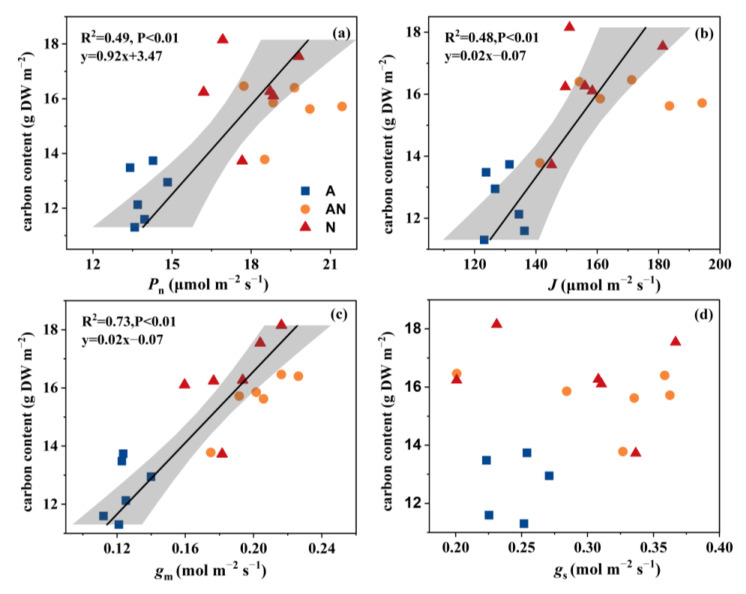
Correlation analyses of the leaf carbon content and net photosynthetic rate (*P*_n_) (**a**), electron transfer rate (ETR) (**b**), mesophyll conductance (*g*_m_) (**c**), and stomatal conductance (*g*_s_) (**d**). A (blue squares), AN (orange circles), and N (red triangles) in the plot represent the sole NH_4_^+^ supply, mixed N supply and sole NO_3_^−^ supply, respectively. The data were fitted by linear regressions.

In comparison with NH_4_^+^ treatment, *P*_n_ was relatively higher in NO_3_^−^ and mixed N treatments, with approximately 1.3−fold greater *P*_n_ (Table 2). In agreement with this, the NH_4_^+^−grown plants exhibited an overall depression of stomatal conductance (*g*_s_), electron transfer rate (ETR), and *g*_m_ (Table 2). Correlation analysis indicated a significant and positive correlation between leaf C accumulation and *P*_n_ (R^2^ = 0.49, *p* < 0.01), ETR (R^2^ = 0.48, *p* < 0.01), and *g*_m_ (R^2^ = 0.73, P < 0.01) (Figure 1). Moreover, photorespiration occurs in conjunction with photosynthesis [16]. Here, we used the value of Γ* to assess the alteration in leaf photorespiration, and the significantly upregulated Γ* indicated a higher photorespiration rate under the NO_3_^−^ condition (Table 2). Notably, the benign electron transfer rate and CO_2_ assimilation rate suggested a different function of photorespiration in plants grown in nitrate from that of plants exposed to stress conditions (Table 2).

### 2.2. Metabolomic Profile Analyses

**Figure 2:**
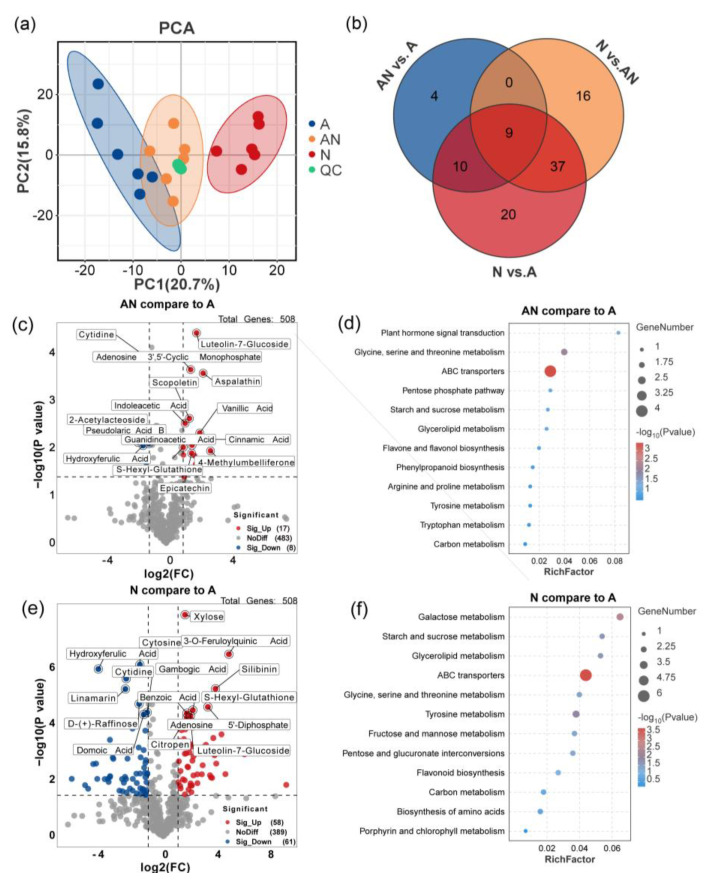
Characterization and analysis of metabolomic profiles of *L. japonica* under different nitrogen sources. Principal component analyses (PCAs) of all detected peaks under different N forms conducted by Liquid Chromatograph Mass Spectrometry/Mass Spectrometry (LC−MS/MS) (**a**). Venn diagram showed the overlap of the differential expressed metabolites (DEMs) in treatment AN versus A, treatment N versus A, and treatment N versus AN (**b**). Volcano plot of metabolomic profiles in the comparison of treatment AN and A (**c**) and treatment N and A (**e**). The red dots denote upregulated metabolites, the blue dots represent downregulated metabolites, while the grey dots correspond to metabolites detected but not significantly changed. Bubble plot of KEGG enrichment analysis of the DEMs in treatment AN versus A (**d**) and treatment N versus A (**f**). The size of bubble represents the number of DEMs enriched in the corresponding pathway and the color of the bubble denotes the significance level of the enrichment analysis, the red color indicates the pathway was significantly enriched, while the blue color revealed the pathways that were not that significant between the treatments. A, AN, and N represent the sole NH_4_^+^ supply, mixed N supply, and sole NO_3_^−^ supply, respectively.

The impact of the form of N on leaf metabolic profiles was investigated using non−targeted LC−MS analysis, and 509 metabolites were detected and mapped. An unsupervised PCA of overall metabolomic profiles revealed clear divisions among the three treatments (Figure 2a). Moreover, plants grown with NH_4_^+^ exhibited a higher similarity to plants grown under mixed N conditions compared with those grown with NO_3_^−^. A Venn diagram revealed that a total of 76 DEMs were identified under NO_3_^−^ treatment compared with NH_4_^+^ treatment, while 23 and 62 DEMs were detected in the comparison of mixed N and NH_4_^+^ and NO_3_^−^, respectively (Figure 2b).

### 2.3. Key Differential Metabolites and Pathways

Taking sole NH_4_^+^ treatment as the control treatment, mixed N and NO_3_^−^ treatments significantly induced the synthesis of phenolic compounds of *L. japonica* (Figure 2c,e). Specifically, replacing ammonium with nitrate led to a gradual increment in 4−methylumbelliferone, cinnamic acid, vanillic acid, aspalathin, and peonidin−3−O−glucoside (Tables S3 and S4). Moreover, dramatic and exclusive increments in the relative contents of N−formylmethionine, caffeoylquinic acid, and 3−O−feruloylquinic acid were observed in NO_3_^−^ treatment compared to NH_4_^+^, by factors of 308, 120, and 21, respectively (Table S3). In contrast, the contents of hydroxyferulic acid, linamarin, and cytidine were significantly decreased under mixed N and NO_3_^−^ treatments.

KEGG enrichment analysis revealed that, among the 23 DEMs identified from mixed N and NH_4_^+^ treatments, 4 were mapped to ABC transporters, 6 were involved in the C and amino acid metabolism, such as glycine, serine and threonine metabolism, starch and sucrose metabolism, and pentose phosphate pathway, and 10 were involved in the biosynthesis of secondary metabolites, phenylpropanoid pathway, and flavone and flavanol biosynthesis (Figure 2d). Similarly, DEMs between NO_3_^−^ and NH_4_^+^ treatment were mainly involved in ABC transporters, galactose metabolism, starch and sucrose metabolism, flavonoid biosynthesis, and glycine, serine, and threonine metabolism (Figure 2f). Taken together, these results revealed a notable variation and interaction in amino acid metabolism, C metabolism, and phenolic metabolism among different N forms.

### 2.4. Amino Acid Metabolism

**Figure 3:**
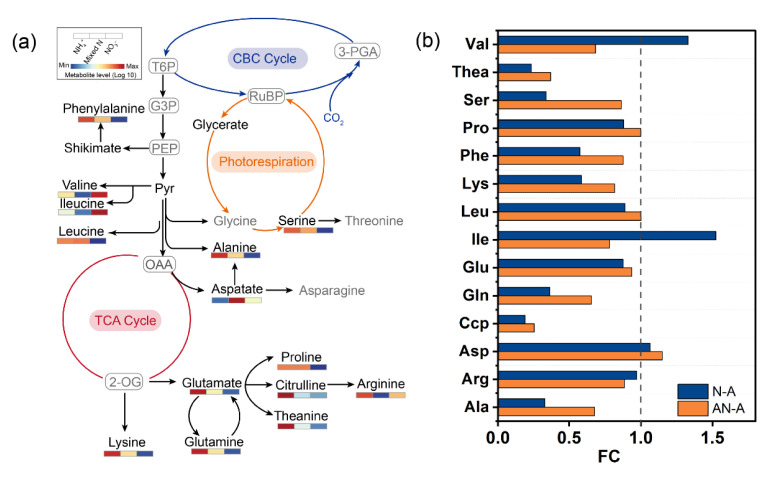
Expression profiles of the amino acid involved in the primary metabolism pathway. The scholar diagram displays the expression pattern of amino acids (**a**). Metabolites in black were present in the pathway, and those in grey were absent from our metabolic profile; the grey letter in the grey box represents the undetected metabolites expected for amino acid. The heatmap corresponding to each amino acid indicates relative expression levels in sole NH_4_^+^ supply, mixed N supply, and sole NO_3_^−^ supply. Relative values of each amino acid content in treatment AN and N compared to treatment A (**b**). T6P, triose−6−phosphate; G3P, glucose−3−phosphate; PEP, phosphoenolpyruvate; 3−PGA, 3−phosphoglycerate; RuBP, ribulose 1,5−bisphosphate; OAA, oxaloacetate; 2−OG, 2−oxoglutarate; FC, fold change; Val, valine; Thea, theanine; Ser, serine; Pro, proline; Phe, phenylalanine; Lys, lysine; Leu, leucine; Ile, isoleucine; Glu, glutamate; Gln, glutamine; Ccp, citrulline; Asp, aspartate; Arg, arginine; Ala, alanine.

**Figure 4:**
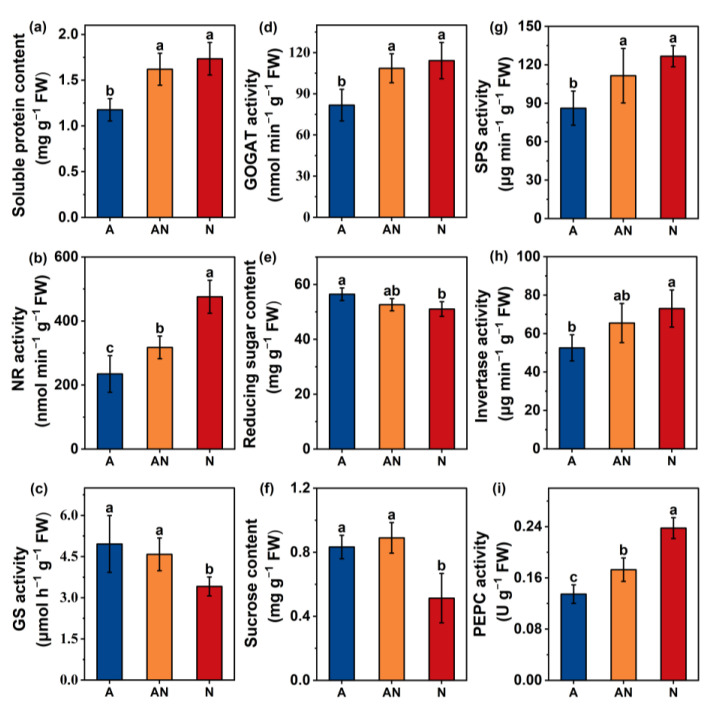
Effects of different N forms on soluble protein content (**a**), nitrate reductase (NR) activity (**b**), glutamine synthetase (GS) activity (**c**), glutamate synthetase (GOGAT) activity (**d**), reducing sugar content (**e**), sucrose content (**f**), sucrose phosphate synthase (SPS) activity (**g**), invertase activity (**h**), and phosphoenolpyruvate carboxylase (PEPC) activity (**i**). A, AN, and N represent treatments with sole NH_4_^+^, mixed N, and sole NO_3_^−^ supply, respectively. Data are means ± SD. Different letters indicate statistically significant differences (*p* < 0.05).

Regarding the detected individual amino acids, most of them showed an increased relative content in plants fed with NH_4_^+^ (Figure 3). Compared with NO_3_^−^ treatment, the content of theanine, serine, glutamine, citrulline, and alanine was substantially higher in NH_4_^+^ conditions, by a relative increment for more than 2−fold; meanwhile, mixed N supply decreased the content of serine and alanine to a lesser extent when compared to NH_4_^+^ treatment (Figure 3b). On the contrary, sole NO_3_^−^ supply induced the increases in valine and isoleucine accumulation by factors of 1.3 and 1.5, respectively. Furthermore, the soluble protein content in plant grown with NO_3_^−^ exhibited a notable elevation in comparison with mixed N and NH_4_^+^ treatment (Figure 4a).

The activities of N assimilation enzymes, including NR, NiR, GS, and GOGAT, were measured (Figure 4). In general, the N form had a significant effect on the activity of the measured enzymes. NR activity progressively increased in plants grown with mixed N and NO_3_^−^ treatments (Figure 4b). GOGAT had a similar pattern, with increased activity under NO_3_^−^ supply (Figure 4c). However, GS activity was, on average, 24% and 31% lower in plants grown with NO_3_^−^ than in plants grown with mixed N and NH_4_^+^, respectively (Figure 4d). NiR activity was not significantly affected by the N source (Figure S2).

### 2.5. C Metabolism

NO_3_^−^ and NH_4_^+^ treatments increased the contents of reducing sugars, especially sucrose, by 10.6% and 62.7%, respectively (Figure 4e,f). SPS, which is a key enzyme in sucrose synthesis, was inhibited by 31.7% under NH_4_^+^ treatment compared with NO_3_^−^ treatment, and by 22.7% compared with mixed N treatment (Figure 4g). The hydrolysis of sucrose into reducing sugar is catalyzed by invertase. Invertase activity was similar in NO_3_^−^ and mixed N treatments, and was, on average, 40.3% and 25% higher than in NH_4_^+^ treatment (Figure 4h). The PEPC enzyme plays a key role in maintaining C assimilation and utilization. In the present study, PEPC activity showed a clear pattern, decreasing with the gradual replacement of NO_3_^−^ with NH_4_^+^ (Figure 4i).

### 2.6. Biosynthesis and Accumulation of Phenolic Metabolites

**Figure 5:**
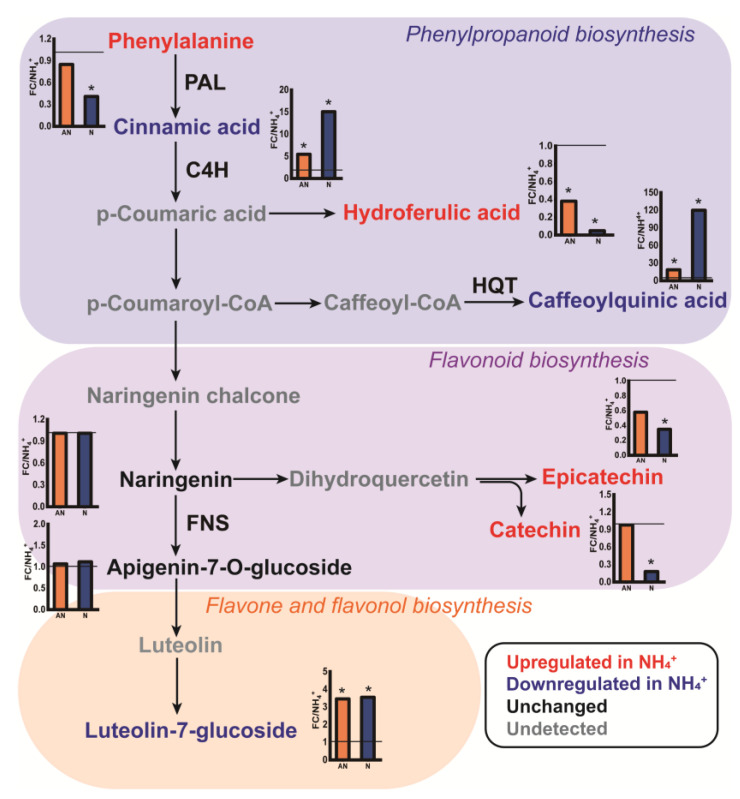
Expression pattern of metabolic profiles and genes involved in phenolic metabolism. Red coloring reflects significantly (*p* < 0.05) increased metabolites under sole NH_4_^+^ condition compared to mixed N and sole NO_3_^−^ condition, while blue indicates the significantly decreased ones. Metabolites in black were present in the pathways but were not significantly changed, and those in grey were absent from our metabolic profile. The green letter denotes the relative key enzymes involved in the pathway. The column plot shows the relative value of each metabolite in treatments AN and N compared to treatment A. Asterisks indicate values determined to be significantly different (*p* < 0.05) between treatment AN, N and A. PAL, phenylalanine ammonia−lyase; C4H, cinnamate 4−hydroxylase; HQT, hydroxycinnamoyl–CoA quinate transferase; FNS, flavone synthase.

Altering the N source significantly affected the pattern of phenolic metabolism in *L. japonica* (Figure 5). Importantly, the relative contents of cinnamic and caffeoylquinic acids were largely increased by factors of 15 and 120, respectively, by NO_3_^−^ supply (Figure 5, Tables S3 and S4). According to the Chinese pharmacopoeia, caffeoylquinic acid derivatives, including CGA, 3,5−di−CQA, and 4,5−di−CQA, are the index compositions representing the medicinal quality of *L. japonica*. In the present study, similar increases in the contents of CGA, 3,5−di−CQA, and 4,5−di−CQA were observed in plants grown with NO_3_^−^ (Figure S2). Luteolin−7−glucoside is a flavonoid that exhibits important pharmacological activity in *L. japonica*. Compared with NH_4_^+^ treatment, mixed N and NO_3_^−^ treatments induced significant accumulation of luteolin−7−glucoside by 3.54−fold and 3.46−fold, respectively, on average (Tables S3 and S4). Importantly, the relative contents of epicatechin, catechin, and (especially) hydro−ferulic acid were increased by sole NH_4_^+^ supply compared with sole NO_3_^−^ supply (Figure 5).

**Figure 6:**
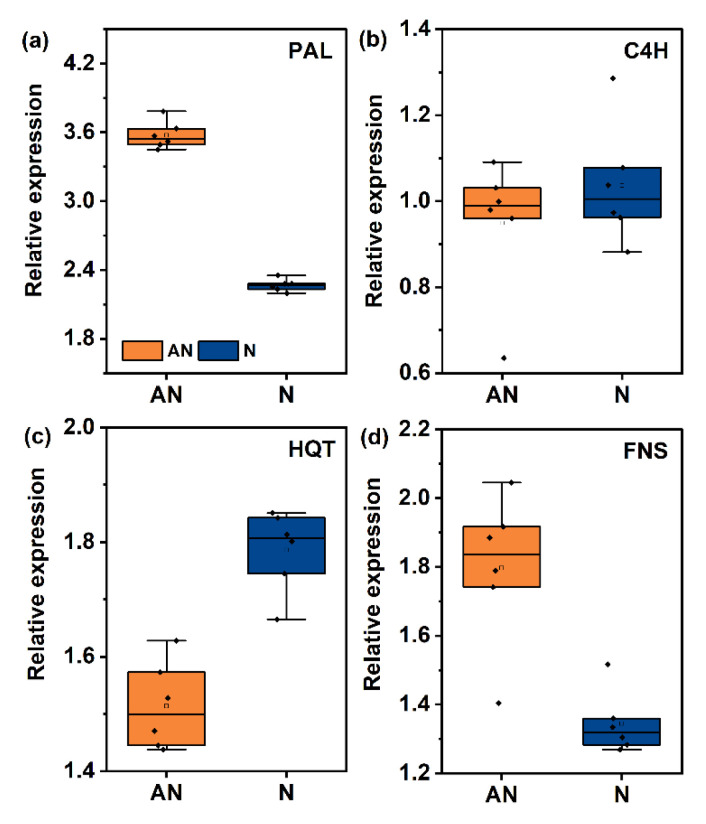
Effects of N form on the relative expression of genes involved in phenolic metabolism pathway. PAL, phenylalanine ammonia−lyase (**a**); C4H, the cinnamate 4−hydroxylase (**b**); HQT, hydroxycinnamoyl–CoA quinate transferase (**c**); FNS, flavone synthase (**d**). The sole NH_4_^+^ supply was set as the control treatment to calculate the relative gene expression levels in treatments AN (mixed N) and N (sole NO_3_^−^ supply).

To determine whether the variation in these metabolites was regulated at the transcription level, we measured the relative expression of key enzymes involved in phenolic metabolism, including PAL, C4H, HQT, and FNS (Figure 6). PAL is the first enzyme in the phenylpropanoid pathway, converting phenylalanine to cinnamic acid. Compared with the sole NH_4_^+^ treatment, the relative expression of PAL was upregulated by 3.6−fold and 2.3−fold in mixed N and sole NO_3_^−^ treatments, respectively (Figure 6a). The C4H enzyme catalyzes the conversion of cinnamic acid to p−coumaric acid. No significant changes were observed in the relative expression of the C4H enzyme (Figure 6b). As the rate−limiting enzyme catalyzing caffeoyl−CoA conversion into caffeoylquinic acid, the relative expression of HQT enzyme increased gradually with the application of NO_3_^−^ (Figure 6c). FNS is the rate−limiting enzyme involved in flavone and flavanol biosynthesis. At the transcription level, FNS expression increased by 1.80−fold under mixed N treatment, and by 1.24−fold under NO_3_^−^ treatment (Figure 6d). Taken together, these results indicate that the relative expression of enzymes involved in phenolic metabolism might be inhibited by sole NH_4_^+^ application.

## 3. Discussion

### 3.1. Photosynthesis and C/N Metabolism in L. japonica in Response to N Source

The applied N sources—NH_4_^+^, mixed N, and NO_3_^−^—exerted a considerable influence on biomass, photosynthesis, and metabolism at a single−leaf level in *L. japonica* (Table 1 and Table 2, Figure 2). NO_3_^−^ supplementation significantly promoted photosynthetic CO_2_ assimilation by *L. japonica*, accompanied by improved C content and C/N ratio (Table 1). Generally, changes in the photosynthetic carbon assimilation rate can regulate metabolic processes by affecting leaf carbohydrate accumulation [14]. Research by Lu et al. [29] showed that carbon starvation mediated by decreased CO_2_ conductance can reduce the biosynthesis of carbohydrates and organic acids. In the present study, correlation analyses showed a strong and positive relationship between leaf C content and *g*_m_ (R^2^ = 0.73, *p* < 0.01, Figure 1), rather than *g*_s_. Moreover, Xu et al. [32] found that the enhanced *g*_m_ largely elevated the C utilization efficiency of *Euonymus japonicus* seedlings. In that condition, plants were forced to adjust their allocation of C and nutrient investments due to the varied *g*_m_ [29]. Along this line, SPS and NI enzymes, which exert in key enzymes coordinating C metabolism in *L. japonica* among different N sources, were evaluated here [33]. The activation of SPS and invertase activities in plants grown with NO_3_^−^ indicated a higher turnover of sucrose and thus more utilization of C (Figure 4) [30]. Furthermore, we observed an increase in the accumulation of sucrose and reducing sugars in plants grown with NH_4_^+^, which could be explained by impaired C utilization by leaves [34].

Metabolic adaptation to N sources has been studied in multiple species and involves N−assimilating enzymes, including mainly NR, NiR, GS, and GOGAT [35,36,37] (Figure 4 and Figure S1). In the present study, NR activity was positively correlated with the proportion of NO_3_^−^ application (Figure 4), similar to a previous study [38]. The GS/GOGAT enzyme system catalyzes the conversion of N from its inorganic form to organic forms, in turn producing a wide variety of amino acids [35]. However, when plants are faced with NH_4_^+^ stress, PEPC enzymes are required to ensure GS/GOGAT carbon demand via regulation of TCA cycle−associated anaplerotic routes [37,39]. In this context, the increase in GOGAT activity under mixed N and NO_3_^−^ treatments are associated with sufficient provision of C skeleton via active C metabolism (Figure 4). Masumoto and Gantt [40] observed the inhibition of GOGAT activity in the chloroplast PEPC gene silencing line *Osppc4*, which further affected C metabolism.

Along this line, *L. japonica* showed a general increment in amino acids in NH_4_^+^ nutrition (Figure 3; Table S3), in accordance with the higher N content compared to mixed N and NO_3_^−^ treatment. At the level of individual amino acids, mainly citrulline and theanine (which are N−rich amino acid) revealed a great accumulation in NH_4_^+^ condition, by relative increments of 5.28−fold and 4.31−fold in relation to NO_3_^−^. Selective preference for NH_4_^+^ in citrulline and theanine production has been confirmed in study by Ruan et al. [41]. Similarly, the relative contents of alanine and glutamine were increased by 3.04−fold and 2.76−fold in NH_4_^+^ treatment compared to NO_3_^−^ (Figure 3). According to Krapp, [42], N−rich amino acids are commonly synthesized to quickly metabolize inorganic N, especially in sole NH_4_^+^−grown plants. Taken together, the results indicate that *L. japonica* can assimilate NH_4_^+^ by enhancing GS activity and accumulating the specialized amino acids as endogenous N storage stores to avoid the toxic effects of NH_4_^+^.

Moreover, contrary to the decrease in amino acid content, soluble protein content in leaves significantly increased under N treatment. Liu et al. [43] observed a similar result in their analysis of apples. Proteins are the main constituents of many enzymes, and their synthesis is initiated from N−formylmethionine [44]. Notably, a significant 308−fold increase in the relative content of N−formylmethionine was observed with N treatment compared with A treatment (Table S3). Although more research is required to determine the mechanism of action, it is most likely associated with the N allocation pattern in plants grown with NO_3_^−^, under which conditions the protein was preferentially synthesized to sustain metabolism.

### 3.2. Phenolic Metabolism Patterns in L. japonica in Response to the N Source

**Figure 7:**
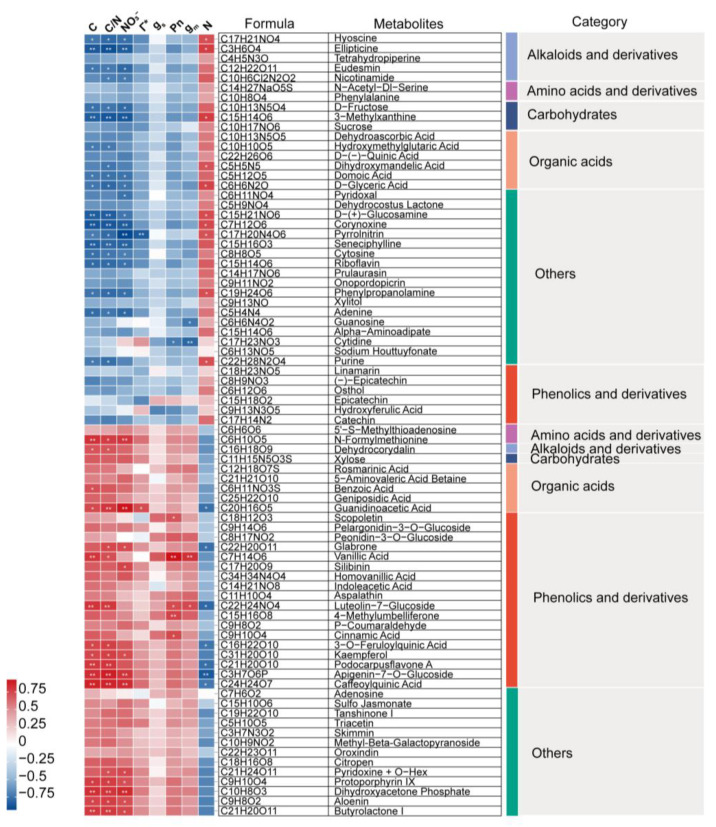
Correlation of DEMs (Tables S3 and S4) with physiological (C, N, C/N, and NO_3_^−^) photosynthetic traits (*P*_n_, *g*_s_, *g*_m_, Γ*). The correlations were estimated by the linear model within 95% confidence level. Colors denote Pearson’s rank correlation between metabolites and traits. Red coloring reflects positive correlation, while blue indicates the negative correlation. The significant correlations are marked with asterisk (*, *p* < 0.05 and R^2^ > 0.70; **, *p* < 0.05 and R^2^ > 0.80).

Phenolic compounds have received considerable attention over the past few decades, since phenolic metabolites have a wide variety of structures and abundance and they have been associated with various beneficial effects on health [45]. The most visible impact of nitrate application on metabolism in the present study was a general increase in the levels of C−rich phenolic metabolites. This pattern of accumulation was most noticeable in phenylpropanoid derivatives (cinnamic acid, caffeoylquinic acid, 3−O−feruloylquinic acid), including coumarin pathway intermediates such as vanillic acid and 4−methylumbelliferone (Tables S3 and S4). The response was especially evident in plants with reduced N uptake. A correlation analysis indicated the potential stimulation of these substances by photosynthesis improvement (Figure 7), confirming earlier observations [30,31]. Kong et al. [46] have also observed the accumulation of phenolic acids, such as ferulic acid, vanillic acid, and caffeic acid, with increased irradiance levels.

Caffeoylquinic acid is a bioactive phenolic substance in *L. japonica* [1,3]. The plasticity of caffeoylquinic acid in plants in response to various environmental situations has been widely reported in recent years [17,47,48]; however, the effect of nitrate on these compounds is still controversial. In the present study, nitrate enhanced the accumulation of caffeoylquinic acid in comparison with ammonium (Figure 5), and a notable and positive relationship between caffeoylquinic acid and C/N ratio and endogenous NO_3_^−^ status was observed (Figure 7). According to Fritz et al. [49], NO_3_^−^ can stimulate phenylpropanoid metabolism by inducing several enzymes involved in the initial steps of the phenylpropanoid pathway. Here, transcriptome profiles showed increased expression of PAL and HQT enzymes in N and AN treatments (Figure 6). These treatments resulted in the accumulation of downstream molecules (cinnamic acid and caffeoylquinic acid), revealing changes in relative gene expression in relation to NO_3_^−^ application. Hydroferulic acid was the most abundant phenolic acid in sole NH_4_^+^-grown plants (Tables S3 and S4). As the precursor of the lignin biosynthesis pathway, its abundance may suggest the promotion of higher lignification by NH_4_^+^. The results were further confirmed by the significantly increased amounts of lignin in plants grown exclusively on NH_4_^+^ (Figure S3). Lignin, the aromatic heteropolymer in plants, is usually synthesized during plant development or stress response [50]. For instance, lignin biosynthesis pathway genes were upregulated under Cu stress, by inducing lignin polymerization enzymes (e.g., peroxidase) [51]. In such cases, NH_4_^+^ may trigger mild oxidative stress, leading to the accumulation of reactive oxygen signaling molecules (such as H_2_O_2_) and activating the lignin biosynthesis. Our findings on NH_4_^+^ are in accordance with those of Kováčik and Klejdus, [17], who suggested this enhanced lignification could serve as a protective mechanism in the presence of exclusive NH_4_^+^ supplement. Taken together, the enhanced lignin biosynthesis could be responsible for the retardation of growth mediated by decreased photosynthesis (Table 1).

Flavonoids, a class of low−molecular−weight phenolic chemicals, are frequently studied due to their variety of biological activities [52]. Many previous studies have observed the effect of NO_3_^−^ on the induction of flavonoid accumulation [53,54]. This response was particularly distinct when nitrate was applied under low levels of N [55]. According to Duan et al. [53], the increased accumulation of flavonoids under NO_3_^−^ supplementation was due to the enhanced C/N ratio and C metabolism. In our study, specific flavonoids in *L. japonica* such as apigenin−7−O−glucoside and especially luteoline−7−glucoside increased in response to C/N ratio under NO_3_^−^ supplement (Figure 7). In accordance with this, the FNS enzyme, which regulates the biosynthesis of luteoline−7−glucoside, was observed to have a higher expression in treatment AN and N in comparison to treatment A (Figure 6). However, a close look may reveal an inconsistent variation because the contents of catechin and epicatechin were considerably decreased in the N and AN treatments compared to treatment A (Figure 7, Tables S3 and S4). Similarly, theanine, the synthetic precursor of catechin biosynthesis, showed a significant decrement in the N and AN treatments (Figure 3). Catechins are constituents of tea−flavor−related compounds; a lot of research on catechins has been reported in tea (*Camellia sinensis* L.) plants, revealing decreased biosynthesis of catechins in a condition of NH_4_^+^ [41,56]. Current evidence has suggested that the inconsistent results on *L. japonica* and tea may be ascribed to the N preference of plants, since tea has a NH_4_^+^ preference. Such variability could also be reflective of the different patterns of C allocation in NH_4_^+^ conditions, which have a low leaf C accumulation [57]. Compared with luteoline−7−glucoside, catechin and epicatechin, when synthesized, are subject to the cost of a lower C skeleton. NH_4_^+^ assimilation requires C skeletons, and sufficient C skeletons could be related to NH_4_^+^ tolerance in vascular plants.

## 4. Materials and Methods

### 4.1. Plant Material and Growth Condition

The experiment was performed at the greenhouse of Nanjing Agricultural University in Nanjing, China. The environmental temperature was set to 26 ± 3 °C in the day and 18 ± 3 °C at night; humidity was set to 50 ± 10%. Photosynthetic photon flux density (PPFD) was maintained at 1000 μmol (photons) m^−2^ s^−1^ at the leaf level with SON−T AGRO 400 W bulbs (Philips, Hamburg, Germany), and the photoperiod was set to 14 h light/10 h dark. One−year−old *Lonicera japonica* plant seedlings with 3–4 leaves were used in this study. For hydroponic culture experiments, seedlings were cultured with a 50% nutrient solution (for composition, see Table S1) for 2 weeks, followed by an adaptation period of the next 2 weeks with a 100% solution. Then, the homogeneous seedlings were transplanted to 12 L plastic containers. Based on our previous experiment, *L. japonica* was treated with different sources of 2.8 mM N: (NH_4_)_2_SO_4_ alone, Ca(NO_3_)_2_ alone, and a combination of 50% (NH_4_)_2_SO_4_ and 50% Ca(NO_3_)_2_. CaCl_2_ was added to the (NH_4_)_2_SO_4_ and mixed N treatments to adjust Ca concentration and dicyandiamide (0.1%) was used as a nitrification inhibitor. The nutrition solution was aerated through the entire culture period and was renewed every 4 days, whereas the pH was adjusted daily to 6.0 ± 0.1 with 1 M HCl and 1 M NaOH.

In the experimental design, there were three experimental units (the plastic box) per treatment and eight subunits (plants) within each experimental unit. Four weeks after the N form treatment, six samples from each treatment were selected for the photosynthetic parameters, gene expression, and metabolomic measurement. The other plants were selected and sampled for the growth and physiological biochemical index.

### 4.2. Gas Exchange and Fluorescence Measurements

A portable LI−6800 photosynthesis system (LI−COR) was used for the measurements of gas exchange and chlorophyll fluorescence parameters on new fully expanded leaves. During determination, photosynthesis was initiated under controlled conditions within the leaf chamber: PPFD, 1000 μmol m^−2^ s^−1^ (10%: 90%, blue light: red light); leaf temperature, 25 °C; CO_2_ concentration, 400 µmol mol^−1^. For fluorescence measurement, an LI−6800 system equipped with a multiphase flash fluorometer chamber was used. The multiphase flash methodology was based on references published by Han et al. [58]. *g*_m_ was calculated as previously described [28]. Six seedlings with similar growth from each treatment were selected for analysis.

### 4.3. Measurement of the CO_2_ Compensation Point in the Absence of Mitochondrial Respiration (Γ*)

The Γ* was measured after dark adaptation (at least 4 h) and six seedlings from each treatment were used for analysis. Prior to the initial measurements, the conditions within the leaf chamber were set at 600 μmol m^−2^ s^−1^ PPFD and 100 µmol mol^−1^ CO_2_ concentration. Leaves were acclimated for 30 min inside the leaf chamber. *P*_n_/*C*_i_ response curves were conducted at a series of CO_2_ concentrations (50, 80, 100 µmol mol^−1^) and PPFDs (150, 300, 600 μmol m^−2^ s^−1^). Their linear regressions were then predicted to converge at one point where the x−axis and y−axis of the point were defined as the photo−compensation point (*C*_i_*) and the day respiration rate (*R*_d_). The Γ* was then calculated according to Sun et al. [59]:

(1) $$\Gamma^{*}={C_{i}}^{*}+\frac{R_{d}}{g_{m}}$$

### 4.4. Measurement of Leaf Area and Biomass

Leaf area was captured using a camera, and the measurement for the leaf area was conducted using IMAGE−PRO plus 6.0 software. The fresh new fully expanded leaf was separated from the seedlings and weighed as leaf fresh biomass (FW). Then, the leaf was dried at 105 °C for 15 min followed by further drying at 65 °C in an oven until the weight was unchanged. The weight was recorded as leaf dry biomass (DW). Leaf water content was calculated as leaf water content (%) = (FW − DW)/FW × 100%. The dry samples were preserved for the determination of later physiological parameters. Six seedlings with similar growth from each treatment were selected for analysis.

### 4.5. Physiology Measurement

Milled dry leaves samples were sieved (<180 μm) and weighed to approximately 10–15 mg to measure leaf C content and N content using an N/C analyzer (multi−EA 5000; Analytikjena, Jena, Germany), and the C/N ratio was calculated accordingly. Sucrose was determined by colorimetric resorcinol method and the reducing sugar was assayed using the dinitro−salicylic acid method. Six replicates from each treatment were selected for analysis.

For NH_4_^+^ determination, samples were homogenized with pre−cooled dilute H_2_SO_4_ (0.3 mM, *w*:*v*, 1:10). The mixture was separated at 15,000× *g* for 20 min, and NH_4_^+^ content was determined according to Sun et al. [59] at a wavelength of 625 nm, with modified phenol−hypochlorite method. For the measurement of NO_3_^−^, 0.50 g samples were homogenized with 5 mL deionized water. The mixture was boiled in water for 30 min, then centrifuged at 12,000× *g* for 20 min to collect the supernatant. NO_3_^−^ content was determined at a wavelength of 410 nm according to the sulfuric acid–salicylic acid method. Enzyme activity, including nitrate reductase (NR), nitrite reductase (NiR), glutamine synthase (GS), glutamate synthase (GOGAT), sucrose phosphate synthase (SPS), phosphoenolpyruvate carboxylase (PEPC), and invertase, was assayed using the kit provided by Comin Biotechnology Co., Ltd. (Huizhou, China). Six replicates from each treatment were selected for analysis.

Metabolites assay was conducted by Agilent 1260 high−performance liquid chromatography (Santa Clara, CA, USA). Leaf samples were soaked in 75% methanol (*w*:*v*, 1:100), and the mixture was sonicated for 30 min and followed by filtration using a syringe (0.45 μm) into a vial. For the determination of chlorogenic acid (CGA), 3,5−di−caffeoylquinic acid (3,5−di−CQA), and 4,5−di−caffeoylquinic acid (4,5−di−CQA), the mobile phases consisted of phase A (0.1% phosphoric acid) and phase B (acetonitrile). The mass spectrometry parameters were set according to Cao et al. [28]. Six replicates from each treatment were selected for analysis.

### 4.6. Metabolomic Analyses

Metabolomic analyses were performed by Bio−Profile Biotechnology Co., Ltd. (Shanghai, China). Firstly, fresh samples (100 mg) were pulverized with liquid N, and a pre−cooled mixture of 200 μL deionized water and 800 μL methanol/acetonitrile (*v*:*v*, 1:1) was added. The mixture was then vortexed and incubated for 1 h. Next, tubes containing the mixture were centrifuged at 6000× *g* and 4 °C for 30 min. The supernatant was carefully transferred into a new centrifuge tube and dried using a vacuum centrifuge. Then, the samples were dissolved in a 100 μL solution consisting of an equal volume of acetonitrile and water followed by another round of centrifugation at 16,000× *g* and 4 °C for 30 min. Finally, the supernatant was collected for ultra−high−performance liquid chromatography using Agilent 1290 Infinity LC system, coupled with AB Sciex triple time−of−flight mass spectrometry 5600 (Framingham, MA, USA). Six replicates from each treatment were selected for analysis.

For metabolite separation, samples were then analyzed using an ACQUIY UPLC BEH column (2.1 mm × 100 mm, 1.7 μm). Gradient elution analysis was performed using a mobile phase consisting of two phases: phase A, 25 mM ammonium acetate, and 25 mM ammonium hydroxide in water; phase B, acetonitrile. The flow rate was set as 0.5 mL min^−1^. After equilibration, each sample was injected with a volume of 5 μL solutions. A gradient profile was employed as follows: from 0 to 0.5 min, the mobile phase was consistent of 95% B; from 0.5 to 7.0 min, the proportion changed gradually from 95% B to 65% B; from 7.0 to 9.0 min, the proportion decreased gradually from 65% B to 40%; from 9.0 to 10.0 min, it remained constant at 40% B; from 10.0 to 12.0 min, the ratio increased from 40% B to 95% B; from 12.0 to 16.0 min, the mobile phase was consistent of 95% B. Acquisition of mass spectrum could be found in the Supplementary Materials (Method S1).

At the end of the assay, the determination of the row data with a unique signal intensity was performed with format conversion, peak alignment retention time correction, and peak area extraction using MS−DIAL software (version 3.98). Accurate mass number matching (molecular weight error < 25 PPM) and secondary spectrogram matching formula were conducted, searching public databases such as HMDB (http://www.hmdb.ca) and Mass Bank (http://www.massbank.jp) on 1 July 2022. Default values (>50% within the treatments) in the extracted peaks were deleted. Then, the positive and negative ion peaks were integrated and applied for pattern recognition with SIMCA−P 14.1 software. Data processing was conducted by Pareto scaling (Par) and the evaluation of the data can be found in the Supplementary Materials (Method S2). A multivariate statistical analysis was performed, which involved unsupervised principal component analysis (PCA) and supervised orthogonal partial least square discriminant analysis (OPLS−DA). For the identification of the most relevant metabolites to the treatments, a threshold of variable importance in the project (VIP) > 1 with a *p* value < 0.05 was set for the selection of differentially expressed metabolites (DEMs).

### 4.7. Total RNA Extraction and qPCR Analysis

Leaf samples were pulverized with liquid N, and the total RNA from each analyzed sample was extracted using Trizol regent (ThermoFisher Scientific, Waltham, MA, USA). The synthesis of cDNA and the analysis of qPCR were performed according to the kit’s manufacturer protocols (Vazyme, Nanjing, China). The qPCR was carried out using Applied Biosystems 7500. Six replicates from each treatment were selected for analysis.

All the procedures were conducted following user protocols. Primer sequences for four phenolic metabolism pathway genes (PAL, phenylalanine ammonia−lyase; FNS, flavone synthase; HQT, hydroxycinnamoyl–CoA quinate transferase; C4H, cinnamate 4−hydroxylase; listed in Table S2) were synthesized by GenScript Technology (Nanjing, China).

### 4.8. Statistical Analyses

All the statistical analyses were performed using SPSS 25.0 software. One−way ANOVA was used to determine the difference between mean values from the treatments, and the significant differences were assessed using the least significant difference test (LSD, *p* < 0.05). Bioinformatic analyses were performed using the OmicStudio tools (https://www.omicstudio.cn/tool, accessed on 1 February 2024). The scatter plot, bar plot, and regression analyses were generated with Origin Pro 2021 software. Image layout was performed using Adobe Illustrator 2021.

## 5. Conclusions

Elevated CO_2_ conductance, especially g_m_, was found to facilitate *P*_n_ and C accumulation in NO_3_^−^−grown plants. Increases in SPS, PEPC, and invertase activities indicated good C utilization in the NO_3_^−^ treatment. On the other hand, NH_4_^+^−grown plants showed increases in carbohydrate and amino acid (with strong increases in citrulline and theanine) synthesis, which may mitigate the ammonium effect. Variations in C accumulation had a significant impact on the levels of C−rich phenolic metabolites. For example, caffeoylquinic acid biosynthesis was mainly activated in plants grown with NO_3_^−^, and was triggered by changes in NO_3_^−^ content and the C/N ratio and mediated by the induction of PAL and HQT enzymes in the biosynthetic pathway, while plants grown with NH_4_^+^−N tended to synthesize fewer C−containing compounds, e.g., catechins and epicatechins. Notably, NH_4_+ may activate the lignin biosynthesis pathway, leading to a significant increase in hydroxyferulic acid and lignin accumulation. In conclusion, nitrate addition results in substantial improvements in the growth and biosynthesis of bioactive components of *L. japonica*. Responses under field conditions need to be further quantified to identify N manipulations that can be targeted for the industrial cultivation of *L. japonica*.

## Figures and Tables

**Table 1:** Effects of nitrogen (N) source on physiological traits of *Lonicera japonica* leaves.

Treatment	Leaf Biomass (g Leaf^−1^ FW)	Leaf Area (cm^2^)	C Content (g m^−2^ DW)	N Content (g m^−2^ DW)	NH_4_^+^ (μmol g^−1^ FW)	NO_3_^−^ (μmol g^−1^ FW)	C/N Ratio
A	0.16 ± 0.02 b	16.6 ± 1.9 a	12.5 ± 1.0 b	1.7 ± 0.1 a	4.9 ± 0.4 a	17.0 ± 1.4 b	7.2 ± 0.2 c
AN	0.22 ± 0.05 a	17.8 ± 2.5 a	15.6 ± 1.0 a	1.8 ± 0.1 a	4.6 ± 0.5 a	18.2 ± 1.5 b	8.7 ± 0.7 b
N	0.22 ± 0.03 a	16.3 ± 1.5 a	16.3 ± 1.5 a	1.5 ± 0.1 b	5.0 ± 0.3 a	25.1 ± 1.1 a	10.7 ± 0.5 a

Note: C, carbon. A, AN, and N represent treatments with NH_4_^+^ alone, a mixed N supply, and NO_3_^−^ alone, respectively. Data are means ± SD. Different letters indicate statistically significant differences (*p* < 0.05).

**Table 2:** Effects of N source on photosynthetic traits of *L. japonica* leaves.

Treatment	*P*_n_ (μmol m^−2^ s^−1^)	*g*_s_( mol m^−2^ s^−1^)	*g*_m_ (mol m^−2^ s^−1^)	ETR (μmol m^−2^ s^−1^)	Γ* (μmol mol^−1^)
A	14.0 ± 0.5 b	0.23 ± 0.04 b	0.12 ± 0.01 c	129 ± 6 b	43.8 ± 0.7 b
AN	19.3 ± 1.5 a	0.35 ± 0.12 a	0.21 ± 0.01 a	168 ± 19 a	43.1 ± 1.0 b
N	18.2 ± 1.3 a	0.29 ± 0.06 ab	0.19 ± 0.01 b	157 ± 13 a	46.2 ± 0.8 a

*P*_n_, saturated photosynthetic rate; *g*_s_, stomatal conductance; *g*_m_, mesophyll conductance; ETR, transpiration rate; Γ*, CO_2_ compensation points in the absence of daytime respiration. A, AN, and N represent treatments with NH_4_^+^ alone, a mixed N supply, and NO_3_^−^ alone, respectively. Data are means ± SD. Different letters indicate statistically significant differences (*p* < 0.05).

## Data Availability

The data underlying this article are available in the article and in its online Supplementary Materials.

## Supplementary Materials

The following supporting information can be downloaded at: https://www.mdpi.com/article/10.3390/ijms26094464/s1.

## Abbreviations

The following abbreviations are used in this manuscript:

C	carbon
CNB	carbon–nutrition balance
C4H	cinnamate 4−hydroxylase
CGA	chlorogenic acids
DEMs	differential expressed metabolites
ETR	electron transfer rate
FC	fold change
FNS	flavone synthase
*g*_m_	mesophyll conductance
GS	glutamine synthetase
GOGAT	glutamate synthetase
HQT	hydroxycinnamoyl–CoA quinate transferase
N	nitrogen
NR	nitrate reductase
NiR	nitrite reductase
OPLS−DA	orthogonal partial least squares discriminant analyses
PAL	phenylalanine ammonia−lyase
PCA	principal component analyses
PEPC	phosphoenolpyruvate carboxylase
*P*_n_	net photosynthetic rates
PPFD	photosynthetic photon flux density
SPS	sucrose phosphate synthase
VIP	variable importance in the project
Γ*	CO_2_ compensation point in the absence of mitochondrial respiration
