# Supplementary material for: Metabolomics Combined with Photosynthetic Analysis Reveals Potential Mechanisms of Phenolic Compound Accumulation in Lonicera japonica Induced by Nitrate Nitrogen Supply
Source: Int J Mol Sci. 2025 May 7;26(9):4464. doi: 10.3390/ijms26094464 (PMC12073090; doi:10.3390/ijms26094464)
Supplement: Supplementary file 1 [file ijms-26-04464-s001.zip › ijms-3595288-supplementary.pdf]

Supplementary data

Title: Metabolomics Combined with Photosynthetic Analysis Reveals Potential Mechanisms of Phenolic Compound Accumulation in Lonicera japonica Induced by Nitrate Nitrogen Supply

Authors: Yiwen Cao, Yating Yang, Zhengwei Tan, Xihan Feng, Zhiyao Tian, Tianheng Liu, Yonghui Pan, Min Wang, Xiaoyu Su, Huizhen Liang*, Shiwei Guo*

Method S1. Acquisition of mass spectrum.

Method S2. Metabolomics data evaluation.

Table S1. Nutrient composition of different nitrogen (N) sources treatments. The pH of nutrient solution was adjusted to 6.0.

Table S2. Primers used for qPCR analyses of leaves of *Lonicera japonica*.

Table S3. Differential metabolites between treatment N and A identified by LC-MS.

Table S4. Differential metabolites between treatment AN and A identified by LC-MS.

Figure S1. Effect of N source on the activity of nitrite reductase (NiR).

Figure S2. Effect of N source on the content of chlorogenic acid (CGA), 3,5-di-caffeoylquinic acid (3,5-di-CQA), and 4,5-di-caffeoylquinic acid (4,5-di-CQA).

Figure S3. Effect of N source on the content of lignin.

Method S1. Acquisition of mass spectrum.

Positive (+) and negative (-) modes of each sample were detected by electrospray ionization (ESI). Samples were separated by UPLC and then analyzed by mass spectrometry using QE Plus mass spectrometer (Thermo Scientific). The ionization was carried out using HESI source, and the ionize conditions were set as follows: Spray Voltage: 3.8 kv (+) and 3.2 kv (-); Capillary Temperature: 320 (±); Sheath Gas: 30 (±); Aux Gas: 5 (±); Probe Heater Temp: 350 (±); S-Lens RF Level: 50. The procedures and settings of mass spectrum acquisition were shown as follows:

Mass spectrum acquisition time was set to 12 min, the parent ion scanning range was set to 80-1200 m/z, the primary mass spectrometry resolution was set to 70,000 @m/z 200, the AGC target was 3e6, the primary maximum IT was 100 ms. Secondary mass spectrometry (MS2 scan) of the 10 highest intensity parent ions was triggered after each full scan, and secondary mass spectrometry resolution were set to 17,500 @m/z 200, the AGC target were set as 1e5, secondary maximum IT were set to 50 ms, MS2 activation type was HCD, isolation window were set to 2 m/z, and the normalized collision energy (Setpped) were set as 27, 29, and 32.

Method S2. Metabolomics data evaluation.

System stability was evaluated and analyzed by comparative analysis of Total Ion Chromatogram (TIC) from quality control (QC). QC samples were obtained from the mixed samples of different treatments. TIC diagrams of QC samples under positive and negative mode were superposed as follows:

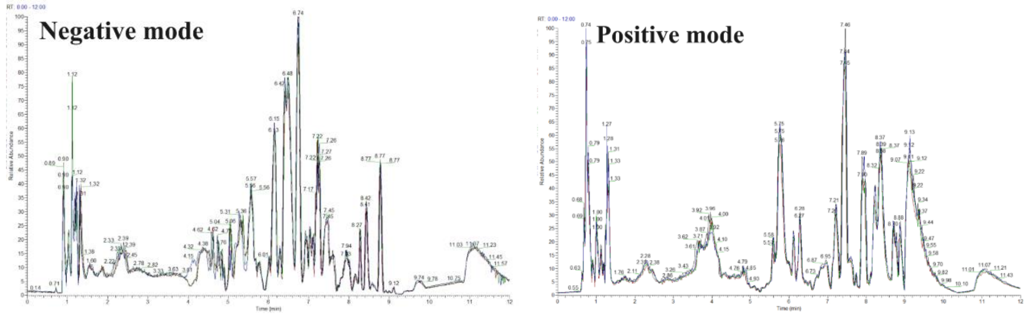

The results showed an overlapped intensity and retention time of each peak, which indicated a lower variation caused by instrument error and a reliable data quality.

Table S1. Nutrient composition of different nitrogen (N) sources treatments.

Compounds	Concentration	A	AN	N
$(NH_4)_2SO_4$	mmol L^{-1}	2.86	1.43	-
$Ca(NO_3)_2 \cdot 4H_2O$	mmol L^{-1}	-	1.43	2.86
$CaCl_2 \cdot 2H_2O$	mmol L^{-1}	2.86	1.43	-
$MgSO_4 \cdot 7H_2O$	mmol L^{-1}	2.00	2.00	2.00
K_2SO_4	mmol L^{-1}	1.03	1.03	1.03
KH_2PO_4	mmol L^{-1}	0.32	0.32	0.32
$MnCl_2 \cdot 4H_2O$	mmol L^{-1}	9.10×10^{-3}	9.10×10^{-3}	9.10×10^{-3}
$(NH_4)_6Mo_7O_{24} \cdot 4H_2O$	mmol L^{-1}	5.2×10^{-4}	5.2×10^{-4}	5.2×10^{-4}
H_3BO_3	mmol L^{-1}	3.7×10^{-2}	3.7×10^{-2}	3.7×10^{-2}
$ZnSO_4 \cdot 7H_2O$	mmol L^{-1}	1.5×10^{-4}	1.5×10^{-4}	1.5×10^{-4}
$CuSO_4 \cdot 5H_2O$	mmol L^{-1}	1.6×10^{-4}	1.6×10^{-4}	1.6×10^{-4}
Fe–EDTA	mmol L^{-1}	3.6×10^{-2}	3.6×10^{-2}	3.6×10^{-2}
DCD	1‰	1.0	1.0	1.0

Table S2. Primers used for qPCR analyses of leaves of *Lonicera japonica*.

Gene	Forward sequence (5’-3’)	Reverse sequence (5’-3’)
PAL	GCTCCTATGTCAAGGCTGCT	TTGTGGTGATGTTCGGAGGG
C4H	GGTTCGGATGGGAGGACGA	GCAACCGCCTTCTCAAAACGA
HQT	CAATCAAGTCCCAAGGCTGT	GGCAGCTAGGACCTCGTATG
FNS	AGGCTAGTGAGGGGGTGAAC	CACCTCACGTACCAATGTCCT
Actin	CGACTACGAGCAAGAACTTGA	CGAACCACCACTAAGCACAA

The expression data were normalized to the Actin gene through the method of $2^{-\Delta\Delta CT}$.

Table S3 Differential metabolites between treatment N and A identified by LC-MS.

Metabolite name	Mode	RT(min)	m/z	VIP	P.value	FC
N-Formylmethionine	Negative	5.86	176.0396	2.46539	0.023495	308.1122
Caffeoylquinic Acid	Negative	5.805	353.0875	11.3471	0.000349	120.0061
3-O-Feruloylquinic Acid	Negative	4.692	367.1039	5.35947	1.71E-06	21.28996
4-Methylumbelliferone	Positive	2.352	177.0546	7.76607	0.000349	17.53366
Cinnamic Acid	Positive	2.347	149.0595	6.4762	0.000595	15.09322
Oroxindin	Negative	1.196	459.0935	1.15777	0.00496	11.80879
Silibinin	Negative	4.65	481.1726	5.93601	2.14E-05	11.40524
Vanillic Acid	Positive	1.3	165.0544	9.74118	0.002691	10.90954
Aspalathin	Negative	2.356	451.124	3.30013	0.000773	10.30822
Peonidin-3-O-Glucoside	Positive	1.649	463.1226	2.02587	0.014051	9.732771
Sulfo Jasmonate	Negative	3.674	305.0706	4.67778	0.013605	9.26854
Pelargonidin-3-O-Glucoside	Positive	1.913	433.112	3.44714	0.011661	8.842445
Guanidinoacetic Acid	Positive	1.302	118.0653	3.92378	0.001506	6.331722
Apigenin-7-O-Glucoside	Positive	2.005	433.1122	3.26737	0.005614	6.280968
Butyrolactone I	Negative	2.209	423.108	3.89595	0.010821	6.045848
Podocarpusflavone A	Positive	1.126	553.1112	1.38558	0.020753	4.923902
Dihydroxyacetone Phosphate	Negative	9.048	168.9985	1.02389	0.010892	4.898249
Aloenin	Positive	3.983	433.112	4.18065	0.000365	4.030195
Protoporphyrin IX	Negative	4.852	561.2546	1.58945	0.000187	3.579038
Luteolin-7-Glucoside	Negative	7.79	447.0935	1.77687	0.000154	3.545893
Kaempferol	Positive	3.903	287.055	2.46576	0.024751	3.544769
Geniposidic Acid	Positive	2.295	392.1538	1.11748	0.006192	3.10215
Benzoic Acid	Positive	6.209	123.0442	1.8699	0.000128	3.013779
Citropen	Positive	6.205	207.065	1.59284	0.000177	3.004283
5-Aminovaleric Acid Betaine	Positive	8.252	160.1332	1.68915	0.000413	2.80164
Xylose	Negative	6.418	149.0444	8.81045	9.58E-08	2.766619
Methyl-Beta-Galactopyranoside	Negative	4.903	193.0711	2.69805	0.002414	2.73605
Homovanillic Acid	Positive	10.947	183.0616	4.99694	0.016821	2.715573
Dehydrocorydalin	Positive	4.872	389.1592	1.20911	0.00044	2.341673
Indoleacetic Acid	Positive	1.038	176.0706	1.15455	0.030598	2.249215
P-Coumaraldehyde	Positive	5.75	149.0599	1.97331	0.008556	2.238042
Skimmin	Negative	6.85	323.0776	1.47521	0.000934	2.101412
Glabrone	Positive	8.028	337.1094	1.20668	0.004195	2.073996
Triacetin	Positive	3.049	218.211	1.58161	0.019361	2.035602
Rosmarinic Acid	Negative	6.4	359.1197	1.25341	0.011885	1.773455
Pyridoxine	Positive	6.804	332.134	2.70886	0.01953	1.668983
Tanshinone I	Positive	7.191	277.0891	1.11769	0.020867	1.641563
5'-S-Methylthioadenosine	Positive	1.419	298.0963	3.46122	0.009057	1.596143
D-Quinic Acid	Negative	7.446	191.0553	10.84	0.013564	0.849757
Sucrose	Negative	7.927	341.11	9.37649	0.006312	0.802974
D-Fructose	Negative	8.354	179.0551	7.91401	0.002437	0.776538
Pyrrolnitrin	Negative	7.343	254.9676	1.04976	0.00221	0.741664
Hydroxymethylglutaric Acid	Negative	6.978	161.0445	6.87573	0.0002	0.620962

Pyridoxal	Positive	2.088	168.0653	2.60782	0.035643	0.610738
Purine	Negative	6.951	119.0338	4.41944	6.67E-05	0.594222
Onopordopicrin	Positive	8.421	371.1463	1.0765	0.02151	0.577074
Prulaurasin	Positive	8.029	296.1126	3.12822	0.004071	0.560997
Alpha-Aminoadipate	Negative	6.176	160.0605	1.78016	0.001317	0.549871
Guanosine	Positive	6.369	284.0983	2.43383	0.000572	0.542071
D-Glyceric Acid	Negative	6.721	105.0179	8.87069	0.000309	0.522221
D-Glucosamine	Positive	6.949	180.0866	1.37973	6.57E-05	0.505491
N-Acetyl-Dl-Serine	Negative	6.766	146.0449	1.84044	0.010604	0.479064
Adenine	Positive	4.421	136.0619	14.3099	0.004711	0.436079
Riboflavin	Positive	5.504	377.145	1.32961	0.013277	0.434801
Ellipticine	Negative	1.375	245.1031	1.61999	0.000456	0.433704
Nicotinamide	Positive	7.839	123.0555	3.8363	0.009731	0.43065
Xylitol	Negative	5.613	151.0602	2.91668	0.006664	0.411643
Phenylalanine	Positive	8.169	166.0864	2.98423	0.037665	0.410066
Domoic Acid	Positive	4.733	312.1432	2.39658	0.000141	0.402922
Dihydroxymandelic Acid	Negative	4.085	183.0234	2.21406	0.031605	0.380086
Epicatechin	Negative	1.389	289.0739	2.06095	0.04548	0.346676
3-Methylxanthine	Negative	7.781	165.0396	4.15127	0.029985	0.344109
Cytosine	Positive	5.942	112.0507	2.41925	3.65E-06	0.337072
Tetrahydropiperine	Positive	6.829	312.1444	2.5765	0.001532	0.319574
Seneciphylline	Positive	1.346	334.1641	1.44641	0.000234	0.252112
Dehydrocostus Lactone	Positive	1.992	248.1643	1.12054	0.035732	0.240427
Corynoxine	Positive	6.168	407.1886	1.12763	0.000236	0.185091
Catechin	Positive	4.962	291.0975	1.41628	0.013086	0.183384
Dehydroascorbic Acid	Negative	3.795	173.0083	10.1281	0.040741	0.181929
Cytidine	Negative	5.943	242.0779	1.48765	1.03E-05	0.178197
Linamarin	Positive	8.374	270.097	2.71936	2.17E-05	0.171998
Hyoscine	Positive	1.314	326.1383	1.29619	0.015396	0.147107
Eudesmin	Positive	5.95	409.1608	1.54068	0.003502	0.098107
Phenylpropanolamine	Positive	3.377	152.107	6.17546	0.015457	0.065644
Hydroxyferulic Acid	Negative	2.291	209.0451	5.89655	5.05E-06	0.048765
Osthol	Positive	4.939	267.0974	1.20529	0.031141	0.030323

Mode, ion mode; RT, retention time (min); m/z, mass to charge ratio; VIP, variable importance in the projection; FC, fold change of treatment N compared to A. A and N indicate the metabolites in NH_4^+ alone and NO_3^- alone supply, respectively.

Table S4 Differential metabolites between treatment AN and A identified by LC-MS.

Metabolite name	Mode	RT(min)	m/z	VIP	P.value	FC
4-Methylumbelliferone	Positive	2.352	177.0546	6.33818	0.015475	6.149598
Cinnamic Acid	Positive	2.347	149.0595	5.47977	0.00918	5.465099
Aspalathin	Negative	2.356	451.124	3.36312	0.000465	4.544988
Vanillic Acid	Positive	1.3	165.0544	8.41808	0.006888	3.960138
Luteolin-7-Glucoside	Negative	7.79	447.0934	1.22676	7.53E-05	3.461843
Peonidin-3-O-Glucoside	Positive	1.649	463.1226	1.64358	0.019249	3.204754
Guanidinoacetic Acid	Positive	1.302	118.0653	3.52107	0.011974	2.88121
Epicatechin	Positive	3.817	291.0836	1.20529	0.017257	2.835835
Scopoletin	Positive	1.952	193.0492	1.42382	0.003579	2.577934
Indoleacetic Acid	Positive	1.038	176.0706	1.99177	0.004439	2.165904
Adenosine	Positive	4.689	268.104	9.00106	0.049935	2.093662
Xylose	Negative	6.418	149.0444	9.3412	0.018608	2.025066
Rosmarinic Acid	Negative	6.4	359.1197	2.35146	0.006159	1.911429
Sucrose	Negative	7.927	341.11	11.3286	0.028022	0.864661
Pyrrolnitrin	Negative	7.343	254.9676	1.42143	0.014533	0.792986
D-Glyceric Acid	Negative	6.721	105.0179	10.4123	0.005031	0.702393
Guanosine	Positive	6.369	284.0983	3.27035	0.004915	0.658913
Sodium Houttuyfonate	Positive	8.34	330.1397	1.42641	0.021143	0.586843
Cytosine	Positive	5.942	112.0507	3.1687	0.000144	0.55055
Linamarin	Positive	8.374	270.097	3.08517	0.00684	0.514553
Cytidine	Negative	5.943	242.0779	1.9976	0.00021	0.407485
Hydroxyferulic Acid	Negative	2.291	209.0451	6.62064	0.012227	0.386425
Hyoscine	Positive	1.314	326.1383	1.86467	0.039385	0.300531

Mode, ion mode; RT, retention time (min); m/z, mass to charge ratio; VIP, variable importance in the projection; FC, fold change of treatment AN compared to A. A and AN indicate the metabolites in NH_4^+ alone and mixed N supply, respectively.

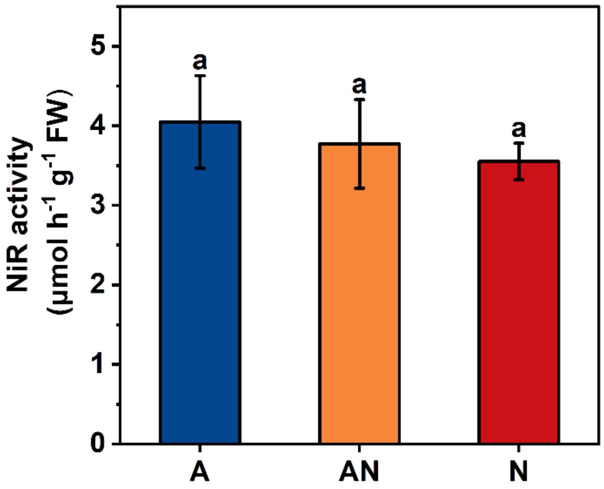

Figure S1. Effect of N source on the activity of nitrite reductase (NiR). A, AN and N indicate the metabolites in NH_4^+ alone, mixed N supply, and NO_3^- alone supply, respectively. Data are means ± SD. Different letters above the bars indicate significant differences at $P < 0.05$.

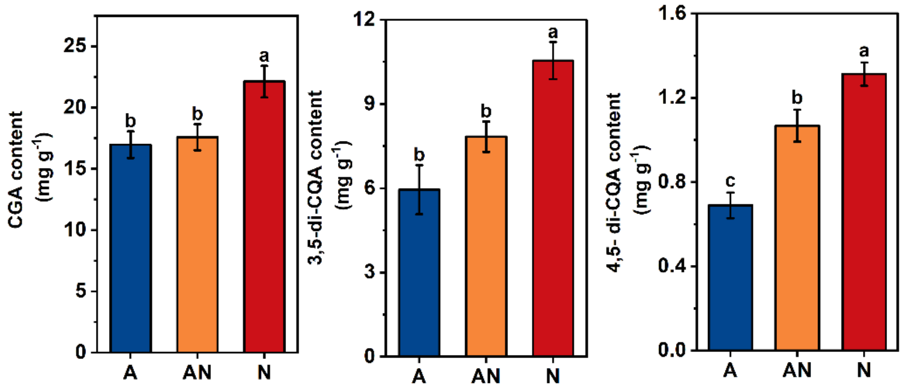

Figure S2. Effect of N source on the content of chlorogenic acid (CGA), 3,5-di- caffeoylquinic acid (3,5-di-CQA), and 4,5-di-caffeoylquinic acid (4,5-di-CQA). A, AN and N indicate the metabolites in NH_4^+ alone, mixed N supply, and NO_3^- alone supply, respectively. Data are means ± SD. Different letters above the bars indicate significant differences at $P < 0.05$.

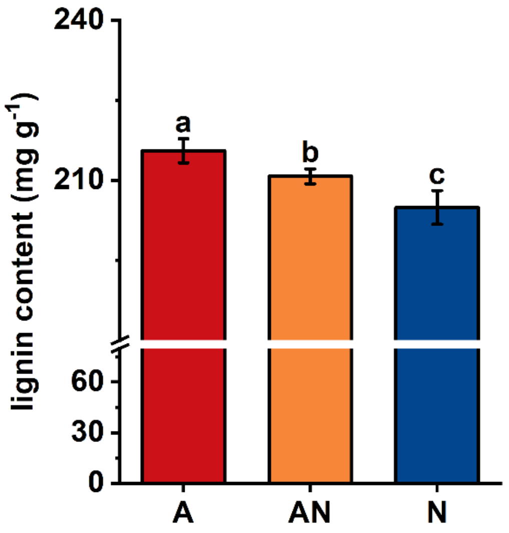

Figure S3. Effect of N source on the content of lignin. A, AN and N indicate the metabolites in NH_4^+ alone, mixed N supply, and NO_3^- alone supply, respectively. Data are means ± SD. Different letters above the bars indicate significant differences at $P < 0.05$.
